# Supplementary material for: Turn-on protein switches for controlling actin binding in cells
Source: Nat Commun. 2024 Jul 11;15:5840. doi: 10.1038/s41467-024-49934-2 (PMC11239668; doi:10.1038/s41467-024-49934-2)
Supplement: Supplementary file 1 — Supplementary Information [file 41467_2024_49934_MOESM1_ESM.pdf]

**Turn-On Protein Switches for Controlling Actin Binding in Cells**

Supplementary Figures

**a**

| | 1 | 10 | 20 | |
|---|---|---|---|---|
| Lifeact | MGVADLIKKF | ESISKEE | | |
| ZO-1 ABS | PAMKPQSVLT | RVKMFENKRS | ASLETKKD | |

**b**

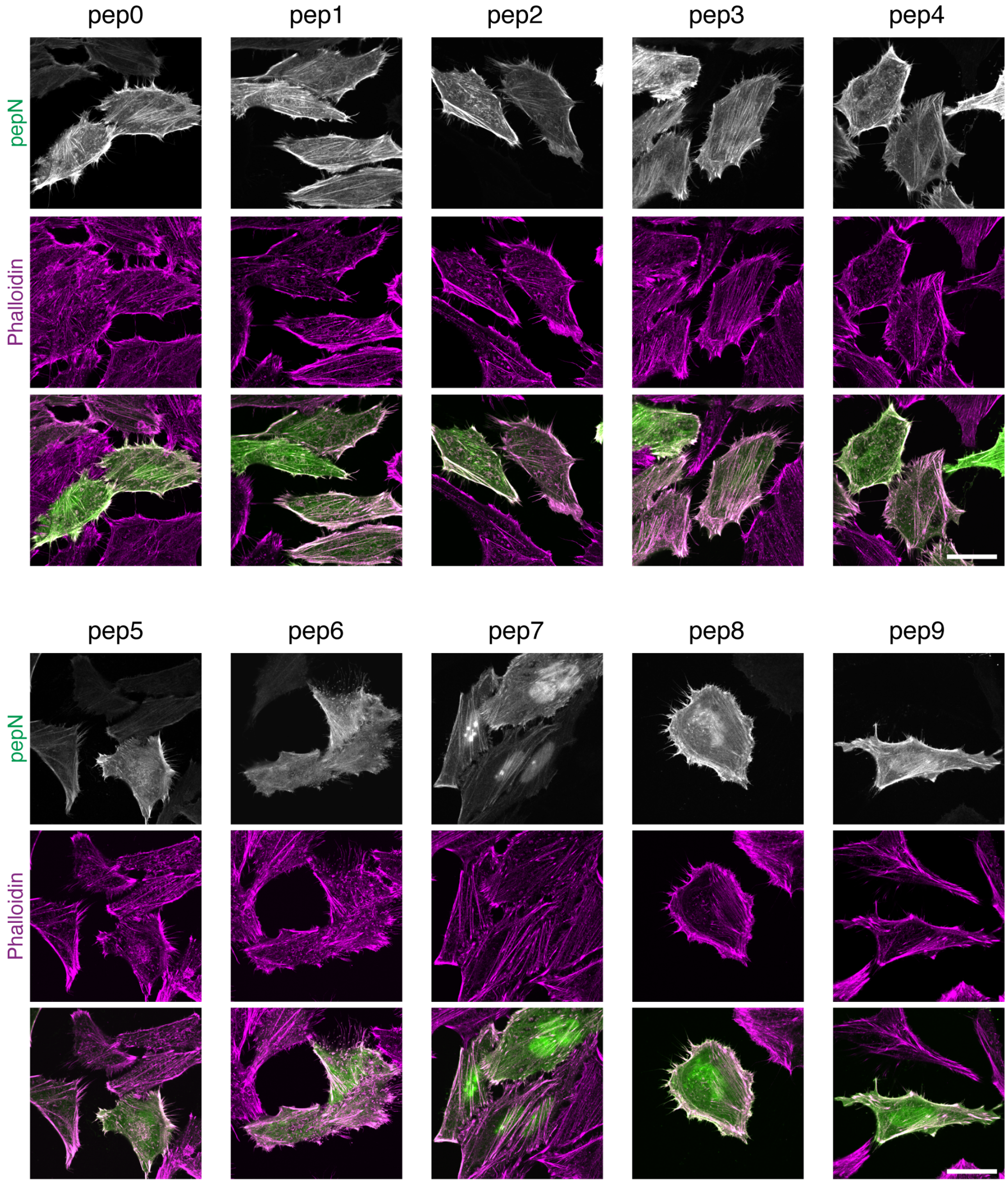


**Supplementary Figure 1. Visualization of ABS-based pepCAST candidates in cells.**
**a,** Amino acid sequences of the ABMs, ZO-1's ABS and Lifeact.
**b,** Fluorescent micrographs of fixed HeLa cells expressing ABS pep0-pep9 in the absence of SZ21. Total cellular F-actin was visualized by Phalloidin staining (magenta). Scale bar, 30 μm.

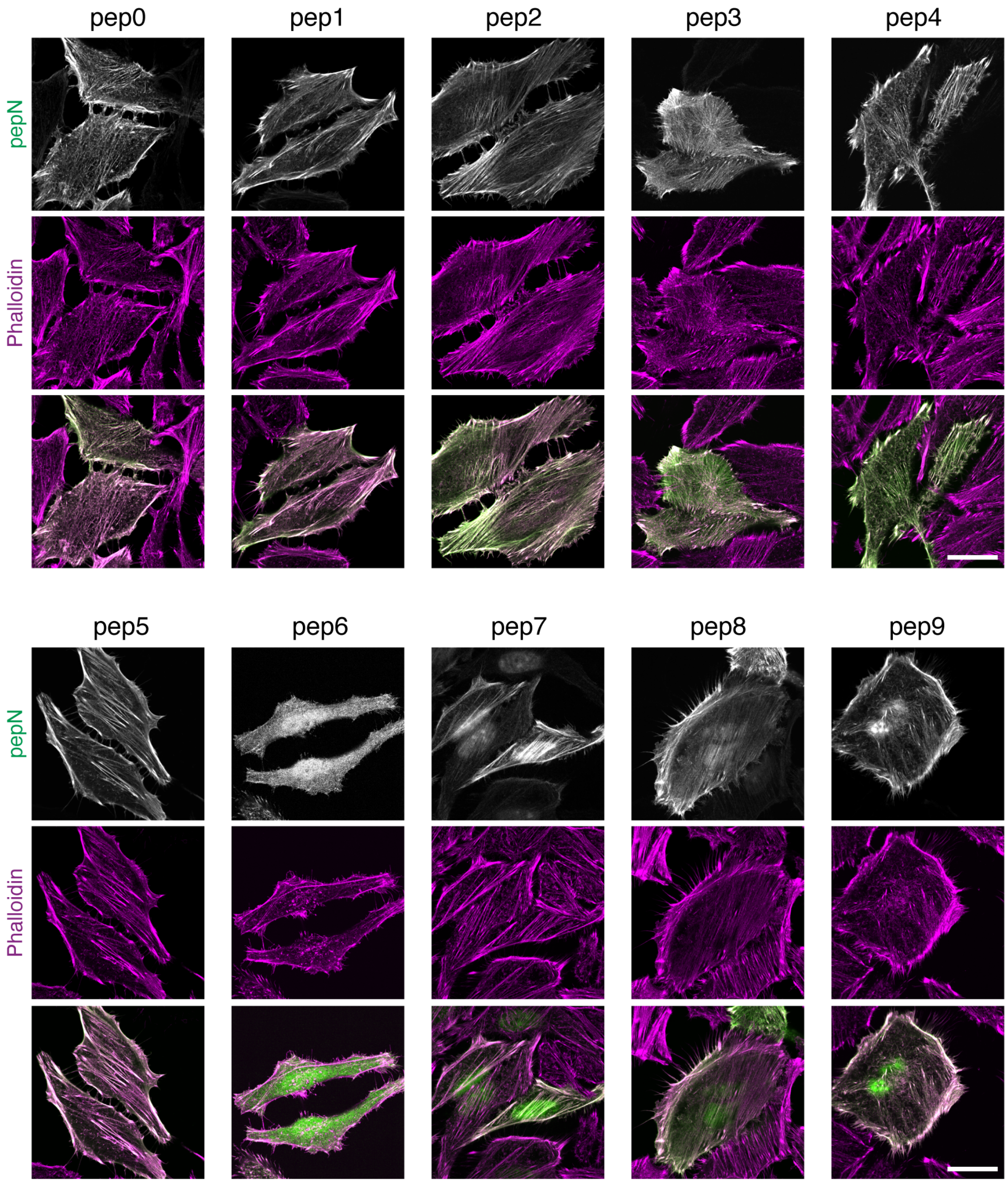


**Supplementary Figure 2. Visualization of Lifeact pep CAST candidates in cells.** Fluorescent micrographs of fixed HeLa cells expressing Lifeact pep0-pep9 in the absence of SZ21. Total cellular F-actin was visualized by Phalloidin staining (magenta). Scale bar, 30 µm.

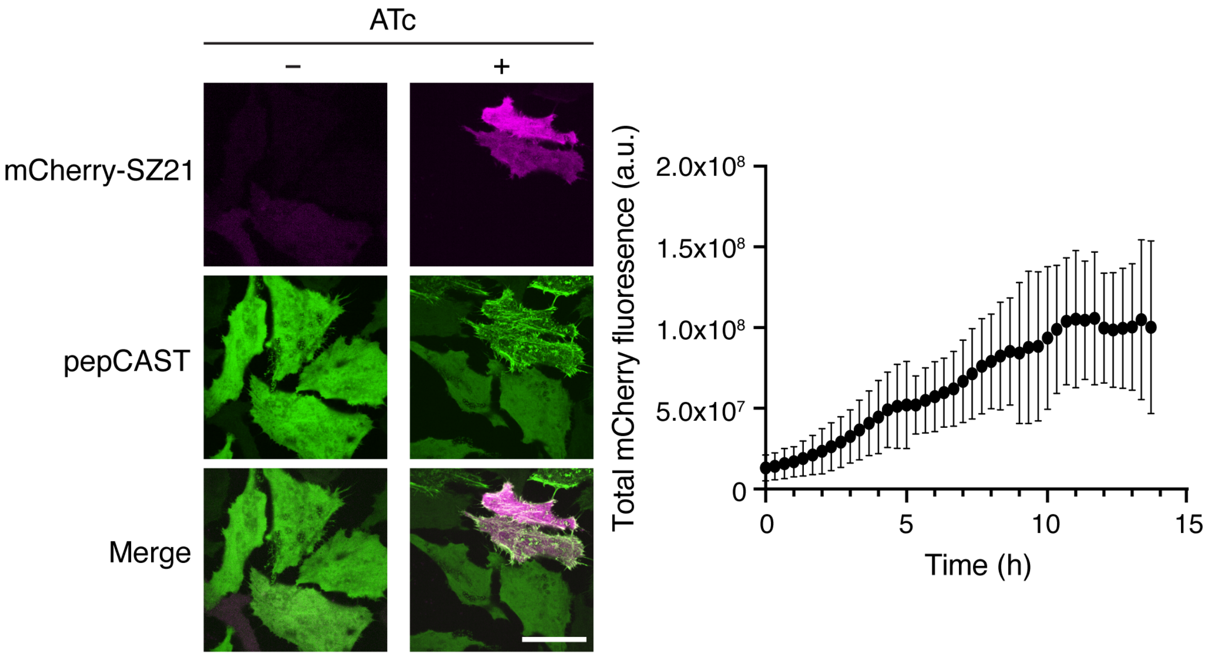


**Supplementary Figure 3. Characterization of SZ21 expression after induction.**
Fluorescent micrographs of live stable pepCAST-expressing HeLa cells transfected with mCherry-SZ21 in a TET inducible plasmid in the absence or presence of 1 μM ATc (left). Fluorescence intensity of mCherry shows increasing SZ21 expression in cells over time following the addition of 1 μM ATc (right). Scale bar, 30 μm. Data are presented as mean ± SD. n = 7 biological replicates. Source data are provided as a Source Data file.

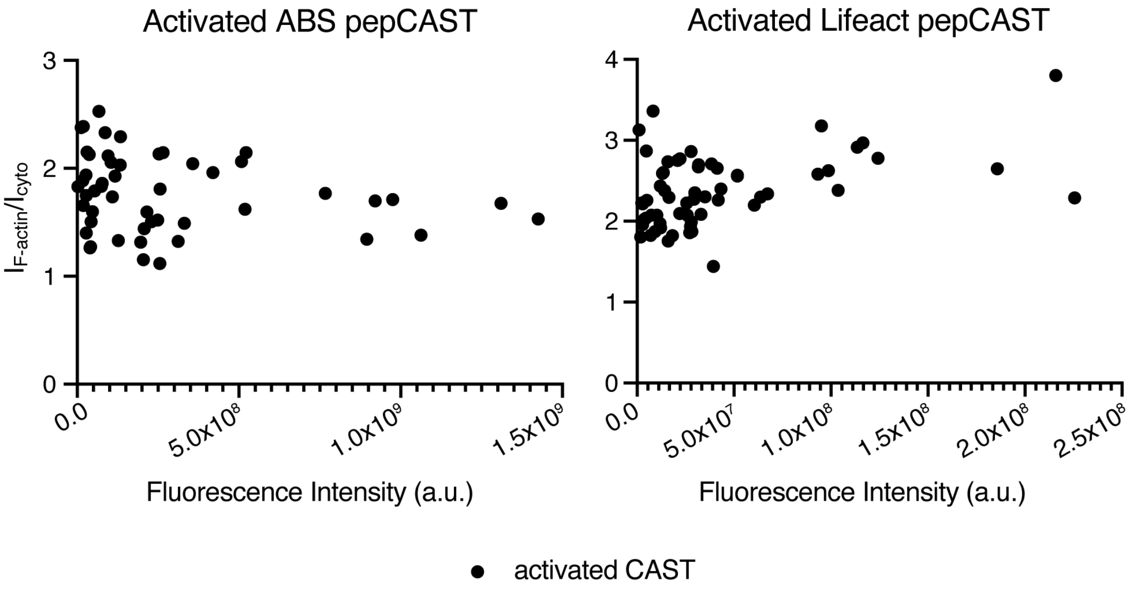


**Supplementary Figure 4**. **Single cell relationship between activated pepCAST F-actin-to-cytoplasmic intensity ratio and pepCAST expression level.**
Calculated ratios of CAST's F-actin-localized fluorescence intensity to its cytoplasmic fluorescence intensity for each cell shows no correlation for activated pepCASTs. n = 50 biological replicates. Source data are provided as a Source Data file.

**a**

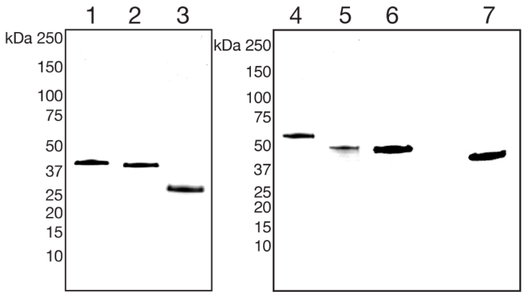


| | Construct | Approximate size |
|---|---|---|
| 1 | ABS pepCAST | 47 kDa |
| 2 | Lifeact pepCAST | 46 kDa |
| 3 | SZ21-mCherry | 34 kDa |
| 4 | ABS smCAST | 55 kDa |
| 5 | ABS optoCAST | 48 kDa |
| 6 | Lifeact optoCAST | 47 kDa |
| 7 | Actin | 42 kDa |

**b**

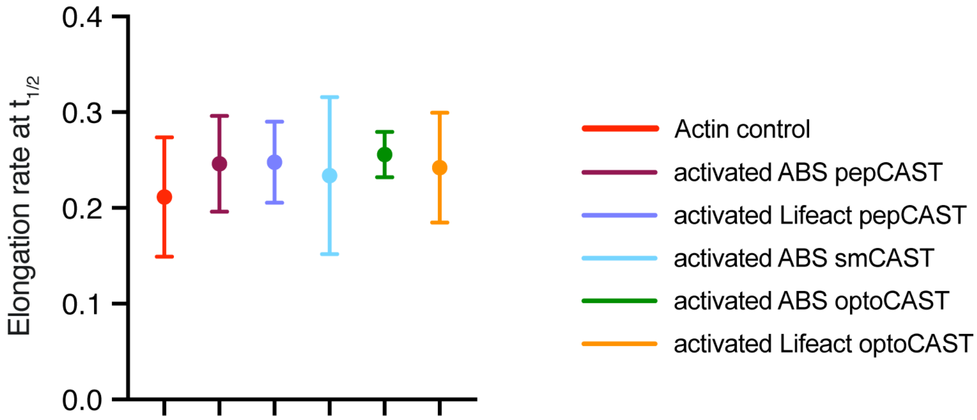


**Supplementary Figure 5**. **CAST purification and actin polymerization assay.**
**a,** SDS-PAGE gels after SEC purification of CAST constructs and G-actin (left) and estimated molecular weights of each protein.
**b,** Quantification of filament elongation rate at $t_{1/2}$ of actin in the absence or presence of activated CASTs.
Data are presented as mean ± SD. n = 5 independent experiments. Source data are provided as a Source Data file.

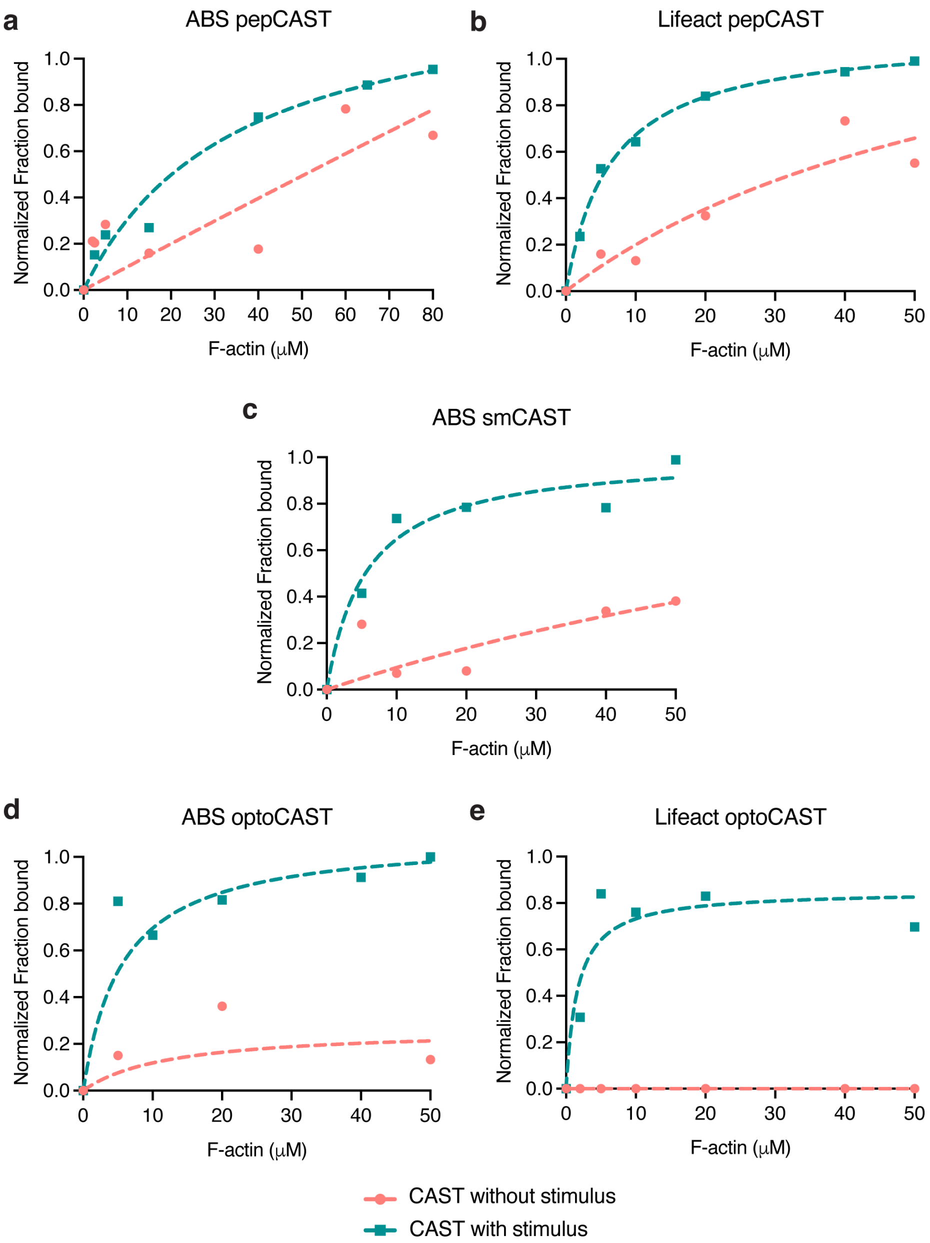


**Supplementary Figure 6. Binding isotherms of CASTs toward F-actin**

**a,** Fraction bound of purified ABS pepCAST in the presence ($K_D$ = 15.44 μM) or absence ($K_D$ = 64.27 μM) of purified SZ21 towards F-actin.

**b,** Fraction bound of purified Lifeact pepCAST in the presence ($K_D$ = 5.02 μM) or absence ($K_D$ = 34.39 μM) of purified SZ21 towards F-actin.

**c,** Fraction bound of purified ABS smCAST in the presence ($K_D$ = 6.07 μM) or absence ($K_D$ = 94.36 μM) of 10 μM Dano towards F-actin.

**d,** Fraction bound of purified ABS optoCAST ($K_D$ = 6.34 μM) or absence ($K_D$ = 206.6 μM) of blue light illumination towards F-actin.

**e,** Fraction bound of purified Lifeact optoCAST ($K_D$ = 2.24 μM) or absence ($K_D$ = n.d.) of blue light illumination towards F-actin.

Circles and squares represent the mean of 2 independent experiments. Curves were fit to a one site – specific binding model (broken lines). Source data are provided as a Source Data file.

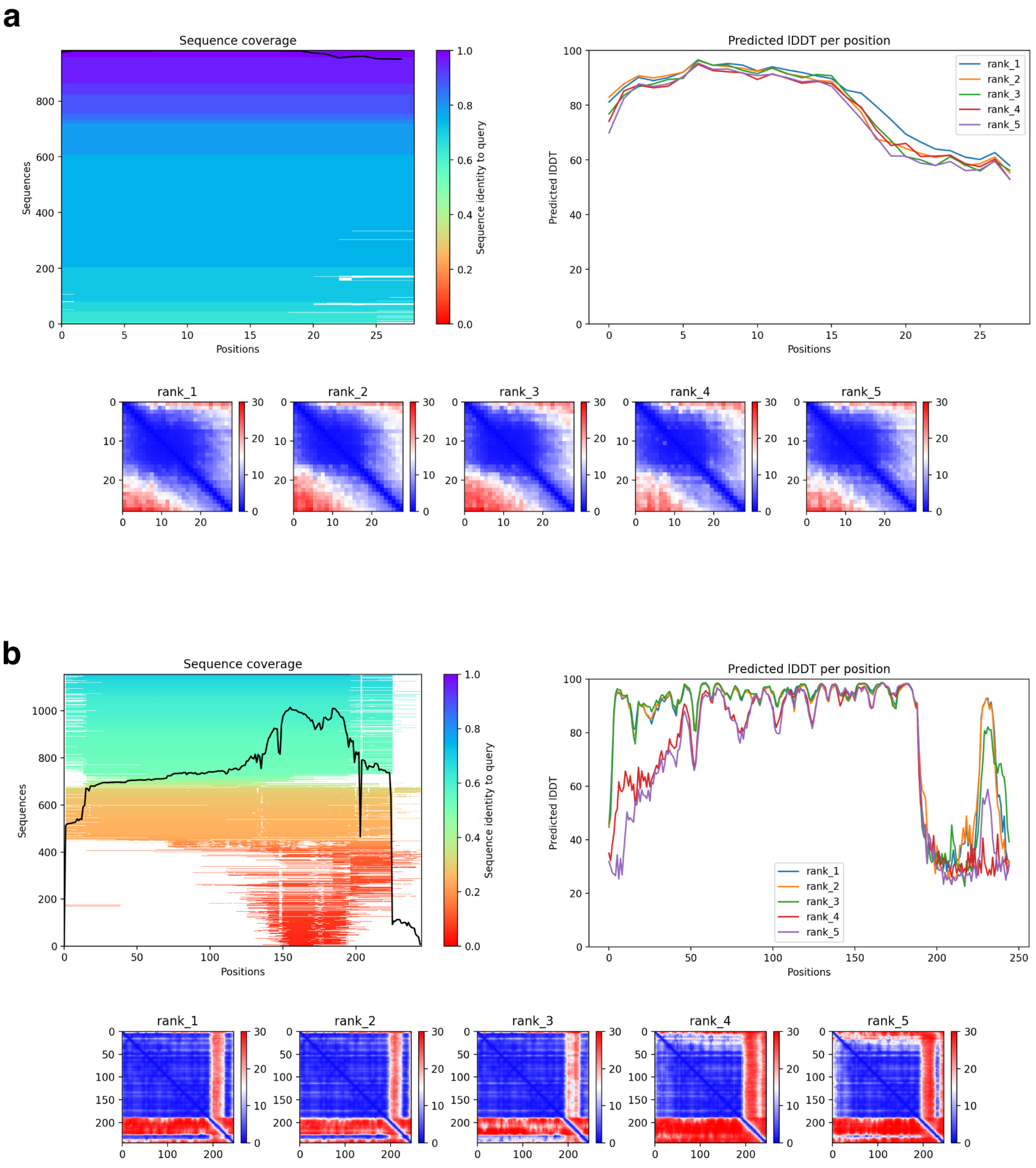


**Supplementary Figure 7. AlphaFold2 structure prediction metrics.**
**a,** Confidence measures for predicted ZO-1 ABS structure showing sequence coverage per residue (top panel, left), local Distance Difference Test (lDDT) scoring (top panel, right), and predicted alignment error (PAE) scoring (bottom panel).
**b,** Confidence measures for predicted smCAST structure showing sequence coverage per residue (top panel, left), local Distance Difference Test (lDDT) scoring (top panel, right), and predicted alignment error (PAE) scoring (bottom panel).
Amino acid sequences used for structure predictions are provided as a Source Data file.

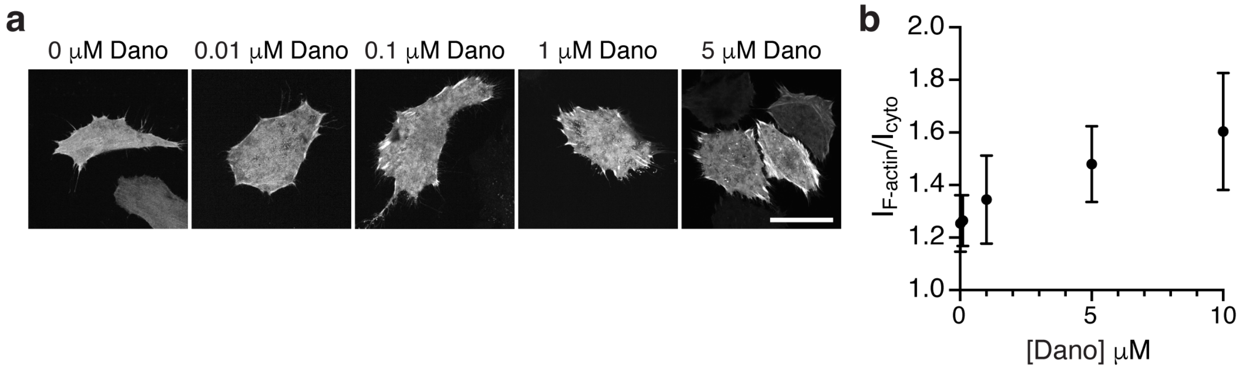


**Supplementary Figure 8**. **Activation of smCAST in cells with different concentrations of small molecule stimulus.**

**a,** Fluorescent micrographs of live HeLa cells expressing smCAST in the presence different concentrations of Danoprevir. Scale bar, 30 μm.

**b,** Quantification of F-actin binding of smCAST at different drug concentrations as the ratio of smCAST's F-actin-localized fluorescence intensity to its cytoplasmic fluorescence intensity. n = 30 replicates. Data are presented as mean ± SD. n = 30 biological replicates. Source data are provided as a Source Data file.

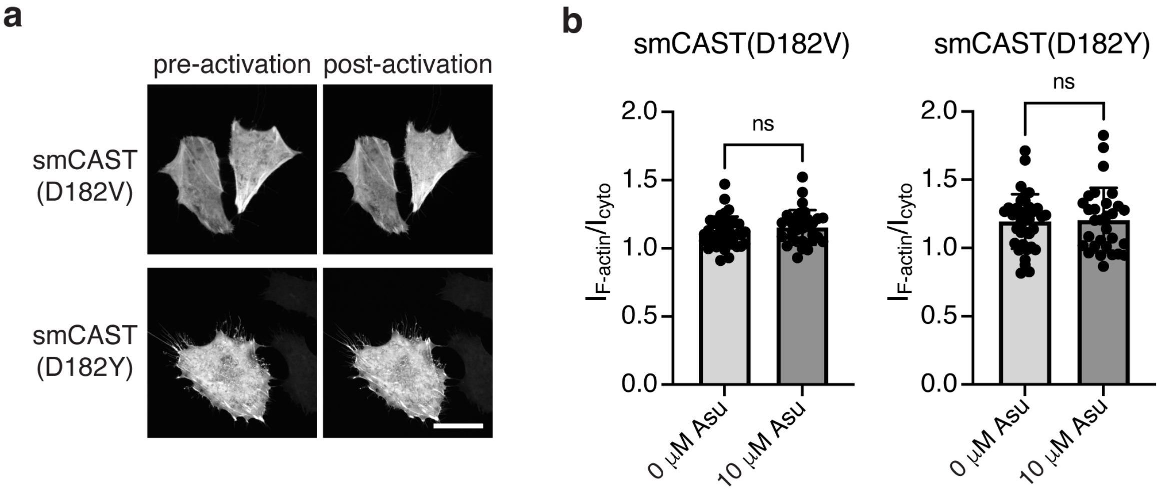


**Supplementary Figure 9**. **Characterization of smCAST mutants in cells.**
**a,** Fluorescent micrographs of live HeLa cells expressing smCAST D182V or smCAST D182Y in the presence or absence of Asunaprevir. Scale bar, 30 µm.
**b,** Quantification of F-actin binding for the smCAST mutants as the ratio of smCAST's F-actin-localized fluorescence intensity to its cytoplasmic fluorescence intensity. n = 30 replicates. Bars represent mean ± SD. P-values were determined using a two-tailed unpaired t-test comparison with 0 µM Asu control. (ns, not significant $P > 0.05$; $^*P < 0.05$; $^{**}P < 0.01$; $^{****}P < 0.0001$). Source data are provided as a Source Data file.

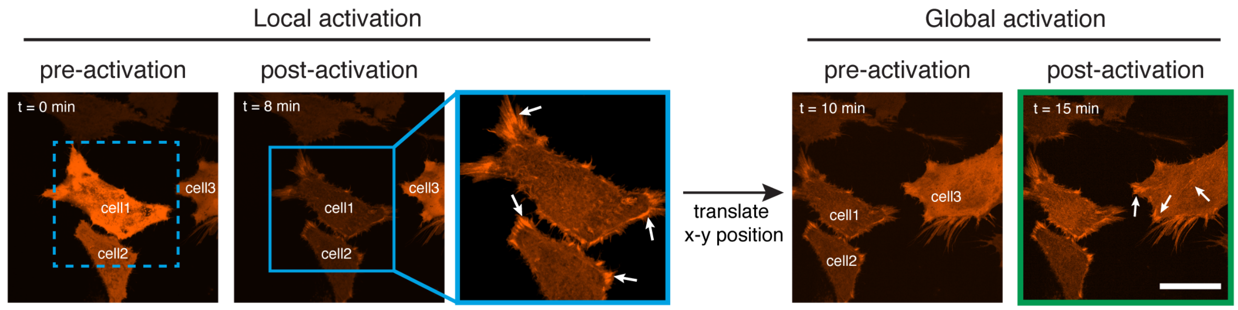


**Supplementary Figure 10. Local optoCAST photoactivation.**
Local Lifeact optoCAST photoactivation. Fluorescent micrographs of Lifeact optoCAST before and after local (blue box) light illumination in live HeLa cells (left). Subsequent global activation (right) of optoCAST shows activation of cell 3, which was excluded from the local region of activation (blue box). Scale bar, 30 µm.

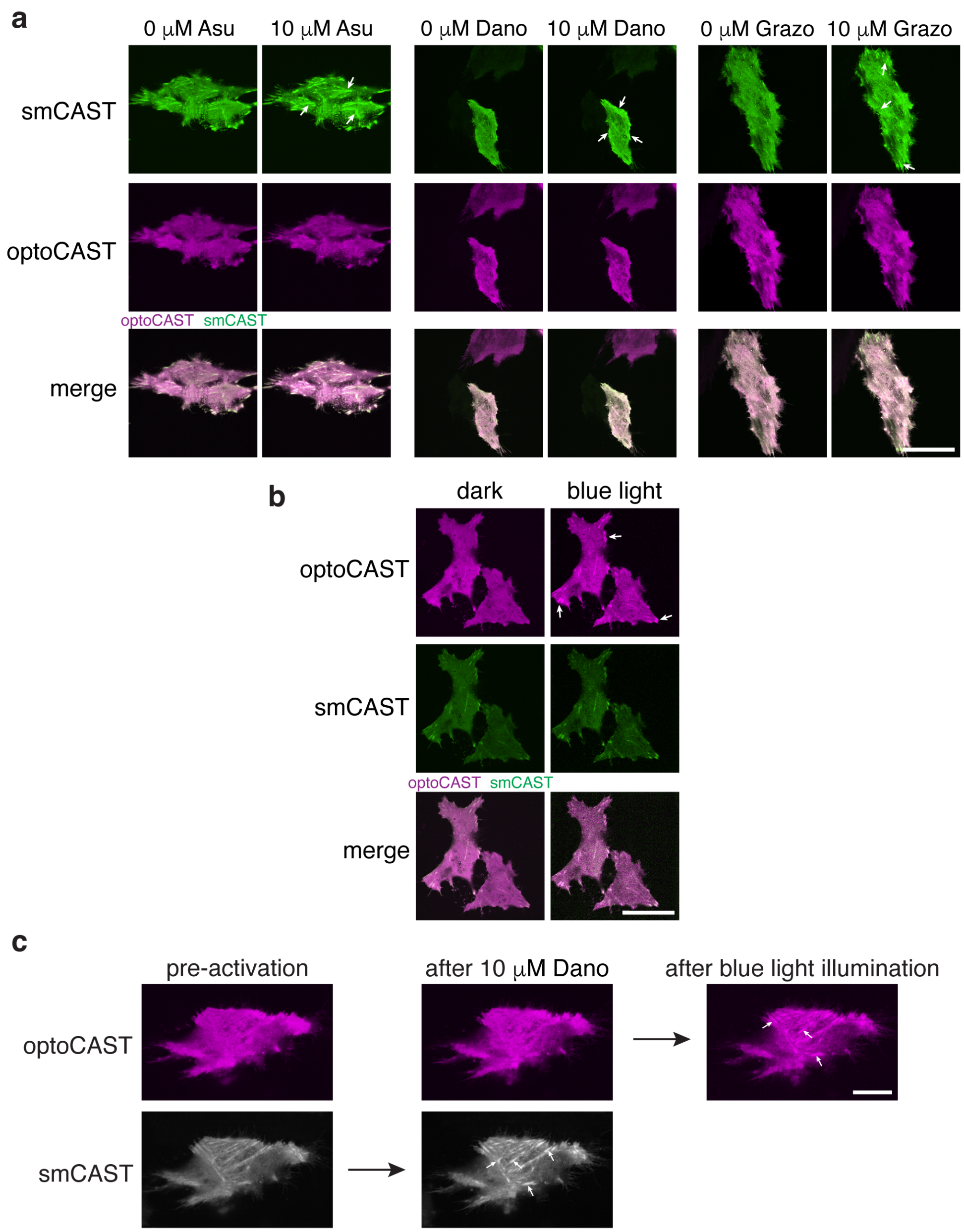


**Supplementary Figure 11. Orthogonal activation of smCAST and optoCAST in cells.**
**a,** Fluorescent micrographs of live HeLa cells co-expressing smCAST and Lifeact optoCAST in the absence or presence of 10 μm Asu, Dano, or Grazo. Only smCAST responds to the small molecule inhibitors and binds to F-actin (white arrows). Scale bar, 30 μm.
**b,** Fluorescent micrographs of live HeLa cells co-expressing smCAST and Lifeact optoCAST in the absence or presence of blue light. Only optoCAST responds to photoactivation and binds to F-actin (white arrows). Scale bar, 30 μm.
**c,** Sequential activation of smCAST and optoCAST in cells. Fluorescent micrographs of a live HeLa cell co-expressing smCAST and Lifeact optoCAST in the absence of blue light and Dano (left). Addition of 10 μM Dano leads to activation of smCAST only (middle panel), as indicated by F-actin binding (white arrows), while optoCAST remains inactive. Subsequent blue light illumination of the cell then leads to photoactivation of optoCAST (right panel), as indicated by F-actin binding (white arrows). Scale bar, 15 μm.

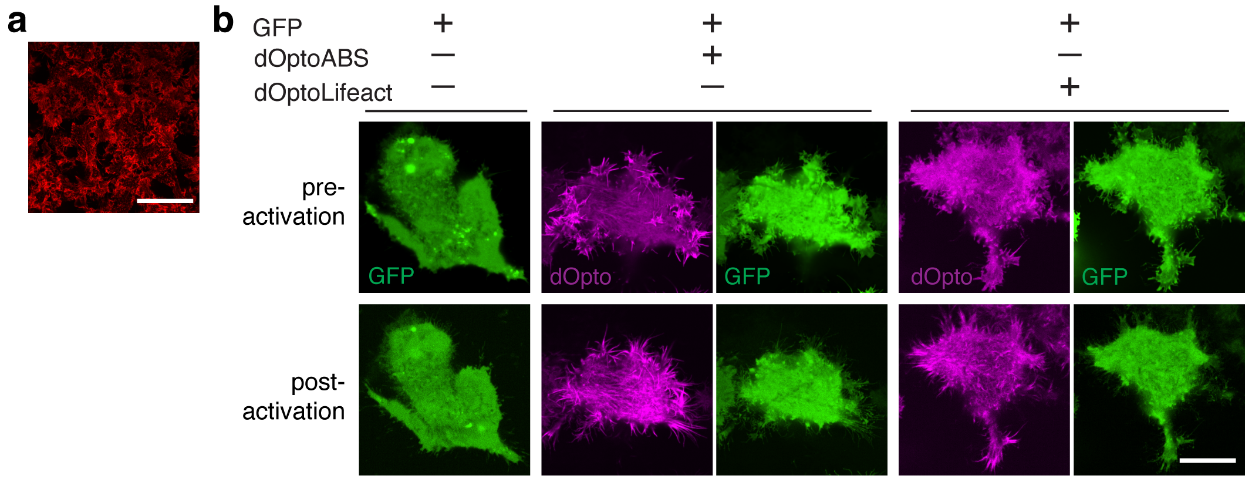


**Supplementary Figure 12. Visualizing cell area in cells expressing dimeric optoCAST.**
**a,** Fluorescent micrograph of HEK 293T cells fixed and stained for endogenous F-actin using Phalloidin. Scale bar, 30 µm.
**b,** Fluorescent micrographs of live HEK 293T cells co-expressing dOptoABS or dOptoLifeact and GFP before and after photoactivation for 10 min. The GFP channel was used for cell area analysis pre- and post-activation. Scale bar, 30 µm.

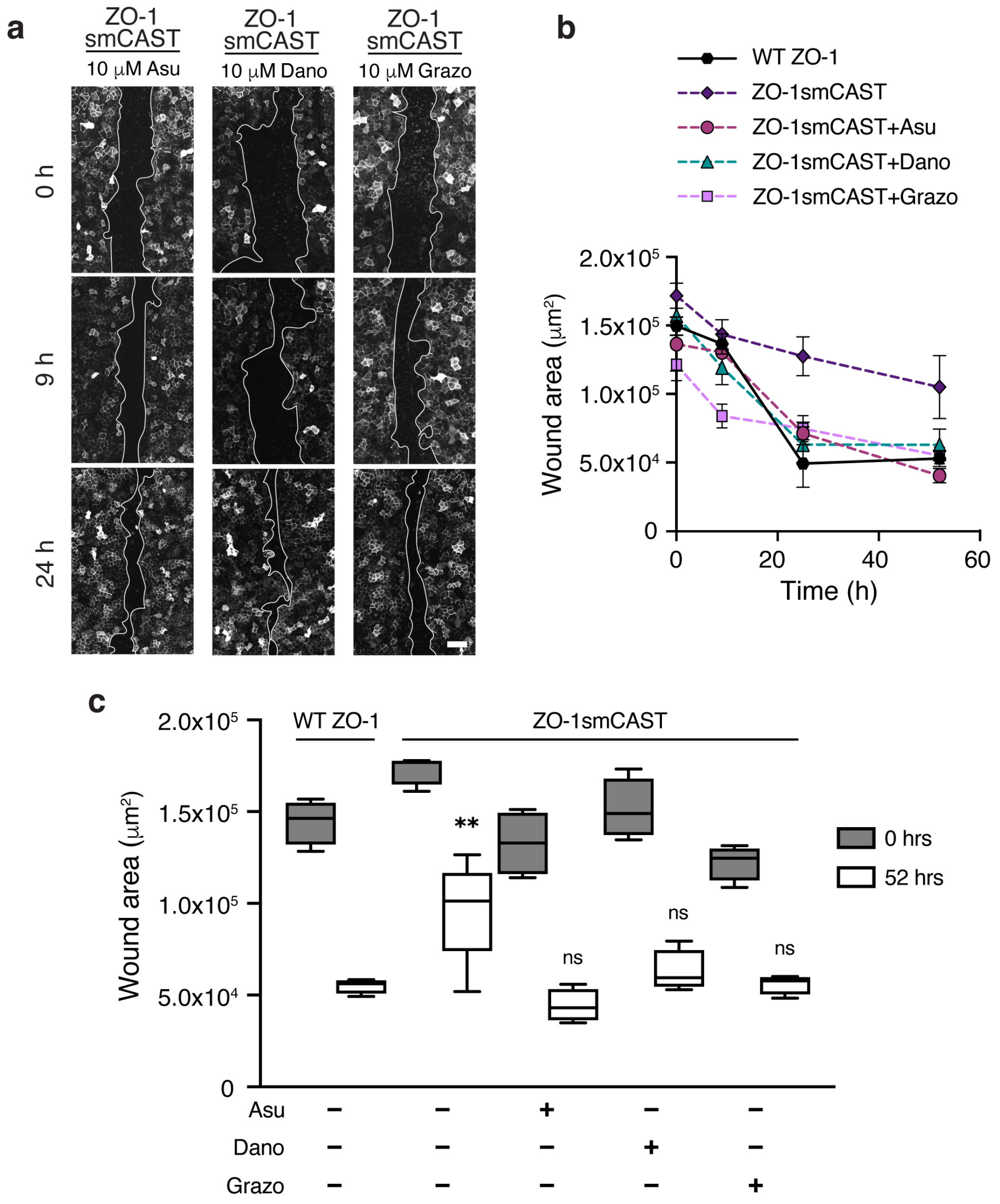


**Supplementary Figure 13. Analysis of collective cell migration with cells expressing CAST-engineered ZO-1.**

**a,** Fluorescent micrographs of collective cell migration in a wounded MDCK II monolayer over time. Stable cell lines lacking ZO proteins and expressing either WT ZO-1 or ZO-1smCAST were imaged following the addition of 10 μm Asu, Dano, or Grazo. Scale bar, 100 μm.

**b,** Quantification of wound area over time. Wound area decreases for cells expressing either WT ZO-1 or ZO-1smCAST and cultured in the absence or presence of 10 μm Asu, Dano, or Grazo. Data are presented as mean ± SD. n = 3 biological replicates.

**c,** Quantification of wound area after 52 h. Box represents 25th to 75th percentiles with the middle line as the median and whiskers as the maximum and minimum values. n = 4 replicates. P-values were determined using a two-tailed unpaired t-test comparison to WT ZO-1 and ZO-1smCAST expressing cells at t = 0 hrs. (ns, not significant $P > 0.05$; $^{*}P < 0.05$; $^{**}P < 0.01$; $^{****}P < 0.0001$). Source data are provided as a Source Data file.

Supplementary Tables

**Supplementary Table 1.** Summary of protein expression conditions for CAST purification.

| Protein | Media | $OD_{600}$ | IPTG (mM) | Expression Temperature | Expression Time |
|---|---|---|---|---|---|
| pepCASTs, SZ21 | Terrific Broth | 0.6 | 1 | 20 °C | 16 h |
| smCAST | Terrific Broth | 0.8 | 0.5 | 18 °C | 18 h |
| optoCASTs | 2x TY | 0.6 | 0.5 | 16 °C | 16 h |

**Supplementary Table 2.** Summary of buffer compositions for CAST purification.

| pepCASTs and SZ21 | smCAST | optoCASTs |
|---|---|---|
| **Lysis Buffer**<br>8 M Urea<br>10 mM Tris-HCl<br>100 mM $Na_2HPO_4$<br>DNase I<br>1 Protease Inhibitor Tablet | **Lysis Buffer**<br>50 mM HEPES<br>100 mM NaCl<br>20 mM Imidazole<br>5 mM DTT<br>20% glycerol<br>DNase I<br>1 Protease Inhibitor Tablet | **Lysis Buffer**<br>20 mM Tris-HCl<br>200 mM NaCl<br>DNase I<br>1 Protease Inhibitor Tablet |
| **Wash Buffer**<br>8 M Urea<br>0.1 mM $Na_2HPO_4$<br>25 mM Imidazole | **Wash Buffer**<br>20 mM Tris-HCl<br>500 mM NaCl<br>20 mM Imidazole<br>10% glycerol | **Wash Buffer**<br>20 mM Tris-HCl<br>200 mM NaCl<br>20 mM Imidazole |
| **Elution Buffer**<br>8 M Urea<br>0.1 mM $Na_2HPO_4$<br>250 mM Imidazole | **Elution Buffer**<br>20 mM Tris-HCl<br>500 mM NaCl<br>300 mM Imidazole<br>10% glycerol | **Elution Buffer**<br>20 mM Tris-HCl<br>200 mM NaCl<br>500 mM Imidazole |
| **Exchange Buffer**<br>50 mM Tris-HCl<br>150 mM NaCl | **Exchange Buffer**<br>20 mM Tris-HCl<br>300 mM NaCl<br>1 mM DTT<br>10% glycerol | **Exchange Buffer**<br>20 mM Tris-HCl<br>20 mM NaCl |
