# Supplementary material for: Turn-on protein switches for controlling actin binding in cells
Source: Nat Commun. 2024 Jul 11;15:5840. doi: 10.1038/s41467-024-49934-2 (PMC11239668; doi:10.1038/s41467-024-49934-2)
Supplement: Supplementary file 10 — Description of Additional Supplementary Files [file 41467_2024_49934_MOESM10_ESM.pdf]

File Name: Supplementary Movie 1
Description: Supplementary Movie file showing pepCAST activation following induction of SZ21 expression. Confocal imaging of a live HeLa cell stably expressing ABS pepCAST and transfected with inducible SZ21. Images were acquired every 20 minutes for 5.5 hours after a 2-hour incubation period of 1 µM ATc to induce SZ21 expression.

File Name: Supplementary Movie 2
Description: Supplementary Movie file showing smCAST activation following addition of small molecule, Grazo. Confocal imaging of a live HeLa cell expressing smCAST. Images were acquired every 5 minutes for 1.5 hours immediately after the addition of 10 µM Grazo.

File Name: Supplementary Movie 3
Description: Supplementary Movie file showing optoCAST photoactivation with blue light. Confocal imaging of a live HeLa cell expressing Lifeact optoCAST. Blue light was pulsed every 2.5 seconds for 6 minutes and images were acquired following each pulse.

File Name: Supplementary Movie 4
Description: Supplementary Movie file showing photoactivation of dimeric optoCAST in single cell. Confocal imaging of live HEK 293T cells expressing dOptoLifeact. Blue light was pulsed every 2.5 seconds for 8 minutes and images were acquired following each pulse.

File Name: Supplementary Movie 5
Description: Supplementary Movie file showing photoactivation of Photoactivation of dimeric optoCAST in tissue island. Confocal imaging of live MDCK II cells expressing dOptoABS. Blue light was pulsed every 2.5 seconds for 8 minutes and images acquired following each pulse.

File Name: Supplementary Movie 6
Description: Supplementary Movie file showing activation and relocalization of ZO-1smCAST. Confocal imaging of live HeLa cells expressing ZO-1smCAST. Images were acquired every 5 minutes for 5.5 hours following a 1-hour incubation of 10 µM Dano.

File Name: Supplementary Data 1
Description: Oligonucleotide sequences used for DNA amplification via PCR
